# Coitus as a Disturbing Element

**Published:** 1897-03

**Authors:** 


### COITUS AS A DISTURBING ELEMENT.

We prefer to preface what we are to say on this subject by laying down some propositions which will probably be generally accepted. Most likely all will agree that married women are more subject to intra-pelvic disease than single women are. Every one with only moderate experience will also have observed that married women respond less readily to treatment than do the unmarried. It is also a fact that the average mother with young children is not in as good health as she was before maternity and that in this regard she is outranked by those of her acquaintances who have not married, or who, at least, if married, have no children. We are also well aware that the added cares of household duties and child's nurse are a tax upon the strength, and that the additional sapping incident to wet nursing makes the mother's lot a still harder one. But all these facts intensify the point we now desire to make.

With the sexual instinct in abeyance, there is saved to the organism, in earlier adult life at least, much energy that is conserved for future emergency or that expends itself in added nutrition, as evidenced by the vigor and bloom of youth. How many women lose this bloom and become relaxed after even a short married life, though conception does not occur, who have rarely known a sick day before marriage? Many are the cases of this kind that come under the notice of physicians.

Many such changes have been traced to the disturbing influence of coitus. The sudden call into full and persistently recurring activity of those previously unexercised parts, causes local congestive and inflammatory troubles, actively maintained by a continuance of the original exciting cause. The suffering woman submits out of a conscientious and even fervid sense of marital duty. The man persists in blissful, but eventually bound to be painful and expensive, ignorance of the damage he is causing. If the physician is eventually consulted for leucorrhea, backache, dragging sensations in the hips, and hypogastrium, and the many other ills peculiar to intra-pelvic troubles, he usually adopts routine treatment, complacently pockets his fee contentedly conscious of duty well performed, whilst his patient does not get well, but is induced by him to bear her sufferings philosophically and with gratitude because she is at least getting no worse. This portrays a condition that most practitioners can readily recall, and that even many patients would not be slow in recognizing.

The local factors causing this misfortune are frictions and associated active disturbance of the relation of the uterus and its adnexa to the pelvic walls and other pelvic contents. To this must also be added the nervous strain of simulating pleasure where there is but pain, and the morbidness caused by secret suffering; for those matters appear of so delicate a nature to a retiring, modest woman, that she is loath to mention them even to her trusted medical adviser. Thus the condition steadily gets worse. A small vagina or a large male organ usually causes trouble, even if gratification is infrequent. Excessive use of these parts is almost always at the expense of general as well as local well-being. The first effects of initial coitus exclusive of the common rupture of the edge of the hymen, is congestion, usually of the vaginal mucous membrane. Frequent repetitions soon involve the uterus and ovaries. These conditions are aggravated by the menses. Then follows a train of ills, the weakest part succumbing first, such as endometritis, versions, flexions, vesical irritability, and ovarian tenderness and congestion. These the physician is called upon to treat.

That removal of the cause constitutes the first requisite to successful and intelligent treatment, is an axiom in medical practice. Few, however, interdict coitus in treating these affections of married women. Yet they cannot help observing the difference in responsiveness to treatment for these conditions in the married and in the unmarried in favor of the latter. Few men even consider the advisability of interdicting coitus till the patient is well, and many of those who do appreciate, at least in part, the desirability of abstinence, refrain from advising it on diplomatic grounds. But this is cowardly, an injustice to the patient, and debasing to the guilty physician. We have repeatedly seen so many good results follow total abstinence from sexual gratification, that it was the only necessary treatment in many instances.

Thus far, allusion has been made only to intra-pelvic trouble caused by coitus; but it is a much greater factor in continuing and intensifying intra-pelvic disease of other origin, and there is where it is most baneful, because much oftener a disturbing element. The lesson to be learned, therefore, from this discussion, is the extreme value of rest of the sexual organs in treating them for congestive or inflammatory trouble. It is not enough, however, to advise the poor suffering woman on this subject, for she is very likely to say nothing about it to her husband, for the double reason that she has a natural delicacy about telling him of conversations upon that subject even with her physician, and she is loath to veto an act to which she conscientiously believes it her marital duty to consent. The most satisfactory plan, from every standpoint, is to spare our patient the indelicacy of the injunction, but to request her to send the husband, to whom the necessity for abstinence can be fully explained, as well as the probable dangers of a disregard of the injunction. If subsequently found to be necessary because of the husband's failure to protect his wife against his own aggressions, she should be sent away to rest whilst undergoing treatment. We affirm that the practitioner who will reason and act upon these suggestions will find many obstinate cases promptly yield, though having failed to respond to any other kind of treatment.—*Medical Council.*

